# Early Childhood Newborn Responsive Parenting Education Program at Maternal Primary Healthcare Facilities of Pakistan and Afghanistan: A Study Protocol

**DOI:** 10.7759/cureus.90864

**Published:** 2025-08-24

**Authors:** Shelina Bhamani, Sanober Nadeem, Sara Sheikh, Misbah Shams, Sehrish Choudhry, Ghulamuddin Delawar

**Affiliations:** 1 Department of Obstetrics and Gynaecology, Aga Khan University, Karachi, PAK; 2 Department of Maternal and Child Health, Aga Khan Health Services, Islamabad, PAK; 3 Department of Obstetrics and Gynaecology, Aga Khan University Hospital, Karachi, PAK; 4 Department of Health Programs and Quality Assurance, Aga Khan Health Services, Kabul, AFG

**Keywords:** early childhood development, feasibility, implementation, newborn responsive caregiving, parenting education program

## Abstract

Introduction: Responsive caregiving is one of the most fundamental pillars of early childhood development. In the newborn stage, the parents are undergoing transitions and require support to understand the nuances of interacting with the young baby. The role of healthcare facilities in this becomes vital as a hub for conducting parenting education sessions that may yield thriving outcomes for young children and their future development. This study aims to explore the impact of the early childhood newborn parenting program on parental confidence and newborn responsive interaction and to explore the perceptions of healthcare providers regarding the intervention implemented in healthcare settings.

Methods: An implementation research design will be used in this study. A total of 500 participants will be included in the study, 400 from Pakistan and 100 from Afghanistan. There are two sites: each site will be randomly assigned as a control or an intervention. The rationale was to ensure that both sites are in different geographical locations. Participants from each side will be chosen using a convenience sampling technique. Cohort 1 (control group) will only be provided with a self-study, one consolidated handout on responsive caregiving, and one-time bed teaching, and will be requested to visit the center after six months (child age) for an endline. While cohort 2 (intervention group) will be provided a six-module intervention consisting of one session at birth with a self-study handout on responsive caregiving, and five monthly sessions (once a month till the infant turns six months) will be conducted. Outcomes measured at the baseline and endline (at six months) include differences in responsive interactions and parenting confidence scores between the control and intervention groups using tools such as Karitine parenting confidence, responsive interaction scale, Caregiver Reported Early Development Instruments (CREDI), WHO QoL, and Patient Health Questionnaire (PHQ).

Results: This is an ongoing study protocol, and the analysis of the results is expected in December 2025.

Discussion: The study will be one of its kind to have been implemented from a healthcare setting, whereby the formal newborn responsive parenting intervention is used, particularly in remote areas of Pakistan and Afghanistan, using a primary healthcare facility for implementation. The study will establish the feasibility of a novel intervention and provide a model of utilization of healthcare settings as hubs for early childhood (ECD)-responsive parenting education. Evidence from this study will be largely applicable to countries with limited resources and countries that are striving to make early childhood parenting education more accessible to the communities.

## Introduction

Early childhood development is fundamental for a sustainable future for all [1-4]. It is crucial to educate parents and caregivers about the importance of early childhood and the domino effect it has on the future [5-7]. Children have an extraordinary capacity to learn, understand, and feel, even without acquiring language and communication skills. The first 1,000 days of life are crucial to the growth and early brain development [8-11]. Neuroplasticity is at its peak with an astounding 1,000 neuronal connections being made per second [12]. As children explore and interact with their surroundings, new synaptic connections are formed, which are further reinforced by positive experiences. Early childhood is a critical period for physical, emotional, and cognitive development, as well as a predictor of future development and skill acquisition. As neuroplasticity declines after the early years, it becomes increasingly hard to offset the effects of toxic stress, neglect, and poor parenting in the future [12]. Today, 250 million children from middle- and low-income countries are unable to reach their development potential [13]. While research has shown that a multitude of interlinking environmental factors, such as immunological, physiological, epigenetic, and psychological factors, influence brain development in a child, a key intervention provided through responsive caregiving can offset the detrimental effects to a significant extent [14]. Hence, parental intervention in this critical time is crucial, as backed by evidence. An early childhood development intervention was introduced in Jamaica for stunted children from low-income families. Children who had received psychosocial stimulation during this period had a 25% increase in earnings and future outcomes [15]. The impact of timely intervention cannot be understated.

Responsive caregiving during the early years lays the foundation for early childhood development and acquisition of essential skills in the future. Responsive and nurturing care provided by primary caregivers can foster brain development, allowing a child to feel a sense of security and safety, build a strong parent-child connection, and strengthen emotional and cognitive growth. A child who has been brought up in a nurturing and responsive environment can easily adapt to the adversity and stresses of life in the future. The importance of responsive parenting in early childhood is emphasized and reiterated by research. A study revealed that preschool children who were provided with responsive caregiving had higher hippocampal volumes. The increase in hippocampal volume is not only indicative of optimal childhood development but also reflects enhanced stress reactivity [16]. Positive experiences provided through nurturing create a virtuous cycle of health, love, and security in children and shape their brains from an early age. According to the life course theory, the early years of life are most sensitive to intervention [17]. The introduction of programs that target caregivers and parents can have a transformative impact on early childhood development. The key to a successful parenting program is educating caretakers about the importance of nurturing care and the far-reaching effects it has on child development. A nurturing environment is sensitive to a child’s needs, provides security and protection, allows for stimulation through play and learning opportunities, and ensures adequate nutrition status and well-being of a child [18]. Research has shown that maternal nurturing care can go as far as attenuating the adverse effects of childhood poverty and adversity [19,20]. Thus, it is important to recognize the power that parenting education interventions can hold.

The UN’s inclusion of early childhood development in the Sustainable Development Goals has resulted in a greater focus on child wellbeing and the introduction of policies and strategies that support this aim [21]. One such intervention that has shown promise is the introduction of parenting programs.

A program that will significantly impact childhood development needs to be multi-sectoral and holistic. While information dissemination through education is essential, teaching parents, especially those who are first-time parents, techniques and strategies to provide childcare is as important. Children thrive with positive attention and focus. Parenting interventions that were initiated in Brazil and Bangladesh had a two-pronged approach with a mix of group and home visits. Both programs showed positive results with enhanced future outcomes, in terms of psychosocial factors and competency [22]. Parenting programs that focus on child enrichment and learning, positive parent-child interactions, and stimulation opportunities for children have the potential to significantly impact development. Practice plays and strategies to provide emotional support to children, as well as caregiving routines, can be taught through these interventions. Moreover, longitudinal follow-ups of parental programs have shown a wide range of beneficial effects and much promise. In children exposed to poverty, the implementation of these programs showed improved health biomarkers [23], intelligence quotients [24], and adult earnings.

In this research, we intend to implement and adapt the model of newborn responsive parenting education in the primary care settings of remote Pakistan and Afghanistan, which we have already implemented in a tertiary care setting [25,26]. This protocol is set to explore the implementation feasibility and fidelity of a structured newborn responsive parenting intervention program for delivered patients. We will examine their parenting confidence, responsive interactions, and experiences of the intervention, and further compare them with the groups who did not receive the full package intervention.

Rationale for Pakistan and Afghanistan

The Foundations for Health and Empowerment (F4HE) project works in several countries, including Afghanistan, Pakistan, India, the Kyrgyz Republic, and Tajikistan. We decided to conduct this project in two countries, i.e., Pakistan and Afghanistan, with some similar geographical and socio-economic features. This will help us to explore the impact of the intervention in two sites, which can then be replicated on a larger scale.

Research objectives

(1) To explore the impact of early childhood newborn parenting education program intervention on parental confidence, newborn responsive interaction, and parental experiences in the intervention group and further compare these with the cohort without intervention.

(2) To explore the perceptions and experiences of healthcare providers regarding newborn parenting education programs in healthcare settings.

## Materials and methods

Study design

**Figure 1:**
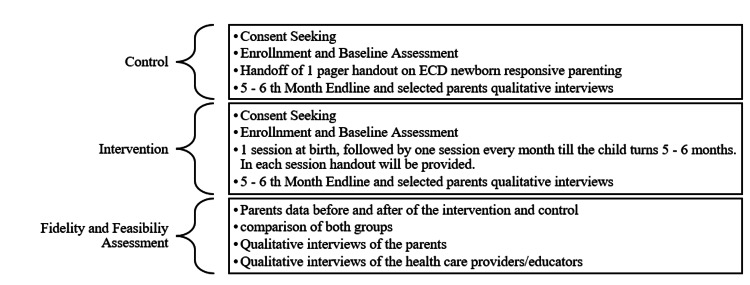
Flow chart of the study procedure

This before-and-after implementation feasibility study will examine the feasibility, fidelity, and adaptation of this intervention in the primary healthcare settings of remote Pakistan and Afghanistan. Implementation research is a relatively new approach in the medical sciences, but it has been widely used in various global, public health, and social sciences research. It also has great potential to allow flexibility for the researchers to be engaged and involved in the process of research. The field sites will be conveniently divided into two groups. The intervention and the control. The intervention group will undergo a structured responsive parenting education program, i.e., six sessions in the first five months of life on a structured curriculum under the supervision of trained lady healthcare workers, while the control group will undergo limited intervention, i.e., a compiled handout at birth for self-study (outlined in Figure 1). 

Study setting

Participants will be recruited from the six basic health centers (BHCs) of not-for-profit private healthcare systems in Pakistan and Afghanistan. These BHCs will be chosen as primary healthcare facilities to pilot the intervention, as shown in Tables 1-2. The BHCs provide maternal, newborn, and child health services (MNCHS), including antenatal care, delivery, and postnatal care (ANC-PNC), family planning, immunization, adult health screening, and regular growth monitoring of <3 years old. Through lady health visitors (LHVs), responsive parenting education will be integrated into their maternal and newborn care model.

**Table 1:** Field sites (Pakistan) F4HE: Foundations for Health and Empowerment

S/n	Site Selection	Location of Health Facility	Type of Health Facility	Level of Services	Average Deliveries Per Month	Covering Under F4HE Geographical Areas
1	Pakistan Intervention Group Site 1 (PIGS 1)	District Upper Chitral	Basic Health Center	Primary	10	Yes
							Pakistan Intervention Group Site 2 (PIGS 2)	District Lower Chitral	Basic Health Center	Primary	4	Yes
	2	Pakistan Control Group Site 1 (PCGS 1)	District Upper Chitral	Basic Health Center	Primary	10							Yes
							Pakistan Control Group Site 2 (PCGS 2)	District Lower Chitral	Basic Health Center	Primary	4	Yes	

**Table 2:** Field sites (Afghanistan) F4HE: Foundations for Health and Empowerment

S/n	Site Selection	Location of Health Facility	Type of Health Facility	Level of Services	Average Deliveries per Month	Covering Under F4HE Geographical Areas
							1	Afghanistan Intervention Group Site 1 (AIGS 1)	Baharak District	District Hospital (DH)	Primary and Secondary	265	Yes
							2	Afghanistan Control Group Site 1 (ACGS 1)	Jurm District	Comprehensive Health Center (CHC)	Primary	77	No

Eligibility criteria

Inclusion criteria for the study enrolment are (i) the newborn’s mother following a normal delivery; (ii) the newborn does not exhibit any underlying morbidities; (iii) the family is not living in ultra-poverty with less than 15,000/- total household income; and (iv) the family can understand Urdu. The exclusion criteria for the study include (i) newborns with underlying conditions or morbidities, (ii) the family living on a 15,000 household income per month, and (iii) mothers with postpartum depression.

Study intervention

The Early Childhood (ECD) Newborn Responsive Caregiving Handbook is a pioneering curriculum designed to support parents and caregivers from the newborn stage up to six months of age. Developed through extensive research into caregiver needs and existing knowledge, the handbook addresses the common gaps and uncertainties many caregivers face during the early months of a child’s life. This comprehensive guide consists of six modules covering key topics: breastfeeding, infant cues and behavior, play, maternal postpartum health, neonatal development, and responsive interactions and attachment. Initially drafted with 12 modules, the content was refined to six core areas based on internal and external expert reviews. The finalized curriculum is freely accessible as a downloadable PDF on the Aga Khan University (AKU) website. Participants in the intervention group receive structured teaching sessions over six months, focusing on each module. Additionally, they are provided with take-home materials to reinforce learning. All interventionists undergo training on the curriculum before field implementation. The links to the study intervention content can be found in the Appendix.

Intervention content

The intervention manual consists of six modules around breastfeeding, infant cues and behavior, play, neonatal development, maternal postpartum health, and parent-child interaction. A comprehensive content coverage is provided in Table 3.

**Table 3:** Intervention content

Title	Content	Concept
Breastfeeding	Exclusive breastfeeding. Importance of skin-to-skin contact. Breastfeeding positions. Breastfeeding problems. Foods for breastfeeding mothers. Hydration in breastfed babies. Breastfeeding vs. formula feeding	Breastfeeding is the best and complete nutrition during the first six months of a child’s life. Breastfeeding and skin-to-skin contact also improve and promote parent-child interaction and attachment. Breastfeeding-related myths
Infant Cues and Behavior	Definition of infant cues and types of infant cues. Definition of infant behavior. Types of infant behavior, verbal and non-verbal communication with infants	Child’s language. Develop an understanding of the caregiver regarding the child’s needs. Caregiver gives sensitive reactions
Play	Power of play, games to play with infants, age-appropriate toys for infants 0-6 months. Toys and play safely for infants. Low-cost, homemade toys, quick tips for parents	Play makes children learn and explore. Engagement of the caregiver with the child during play
Maternal Postpartum Health	Postpartum maternal health. Postpartum blues, mental health checklist for new moms. Impact of maternal mental health on growing children and asking for help. Postnatal exercises and strategies to support maternal and infant mental health	Mother’s mental and physical health is a priority. It’s ok to ask for help during the postpartum phase. Timely referrals
Neonatal Development	Definition of ECD and nurturing care, domains of ECD. Description of neonatal reflexes and types of neonatal reflexes. Development milestones from 0 to 8 weeks and 3 to 6 months. Strategies for effective newborn care, infant massage, immunization, tummy time, waste disposal, and sleep modulation	ECD and holistic development. Important reflexes and developmental delays. Uniqueness of the child. Routine preventive and hygiene practices
Responsive Interactions and Attachment	Definition of attachment. Introduction to parent-child interaction. Benefits of a father’s engagement. Explaining parent-child interaction with the Barnard model (teaching and feeding interactions). Family bond	Parent-child interaction significance. Teaching interactions. Feeding interactions. Fathers’ engagement

Data collection tools

Basic Demographic Information Form

The basic demographic information form is designed to gather information pertinent to delivery outcomes, caregivers' age, education, socio-economic status, household data such as water and sanitation conditions at home, electricity and technology status, etc. This form also entails caregivers' knowledge about emergency contact numbers and child rights. Additionally, the form gathers some specific information about both the newborn and the mother, such as the mother’s height and weight, and the newborn’s gender and birth weight.

Responsive Interaction Scale

The AKU ECD team has developed a short, responsive interaction scale, which is validated in the local population, that helps practitioners explore the interaction between the caregiver and children from birth to six months. The scale is used to classify newborn handling, responsive interactions, consoling techniques, engagement, and distress. This tool can be used in healthcare and community settings to assess responsive parenting and parent-child interaction.

WHO QoL Scale

This scale is used to assess caregivers' overall well-being and quality of life across multiple dimensions, such as physical health, psychological health, social relationships, and environmental factors.

Karitane Parenting Confidence Scale

This scale helps explore caregivers’ confidence in caring for their infants. It is a self-reported questionnaire, and minimal clinical instructions are required to fill it out.

Harvard CREDI

It helps health professionals to track and assess developmental milestones. The CREDI focuses on measuring children’s early skills and behaviors in four primary domains: motor, cognitive, language, and social-emotional development.

PHQ-9

This self-report questionnaire is used to assess the degree of depression and its severity for screening, diagnosis, monitoring, and providing appropriate help according to their need. It comprises nine self-administered questions for easy evaluation based on the Likert scale.

A Semi-structured Interview Guide

A semi-structured interview guide will be used to explore caregivers’ experiences of the healthcare facility's newborn parenting education programs.

Sample size, recruitment, and enrolment

A total of 500 participants will be chosen, 100 from four primary healthcare facilities in the regions of Chitral, Pakistan, and 100 from two primary healthcare facilities in Afghanistan. A total of 200 before-intervention participants as a control group and 200 intervention participants in Pakistan will be recruited. Similarly, for Afghanistan, 50 pre-intervention (control) participants and 50 intervention participants will be recruited as a control group. The sample size is chosen based on convenience sampling, and the facilities are selected considering the number of deliveries and outreach feasibility. It is anticipated that 40% of the total annual deliveries may consent to participate, and based on calculations, this will help us achieve our N for each of the cohorts. Additionally, there might be variations in the actual data collection.

Data collection plan

We will have two cohorts: a cohort before the intervention and an after-cohort with the intervention. Each cohort will last for six months.

Cohort 1 (control group): Baseline data will be collected, and they will be provided with a self-study pictorial module. They will be tracked and requested to visit the center at six months (child age) post-delivery. At six months, their endline data on responsive interactions, parenting confidence, and patient experience will be collected.

Cohort 2 (intervention group): Baseline data will be collected, and they will be provided a six-month intervention consisting of one session at birth with a self-study manual, and five monthly sessions (once a month till the infant turns six months). At six months, they will also be assessed on the measures mentioned above, and endline will be collected.

Data analysis

Both qualitative and quantitative data analysis strategies will be used to conduct data analysis.

Quantitative analysis will be performed using the statistical package for the social sciences (SPSS Inc., Chicago, IL). Descriptive statistics, such as frequencies, percentages, means, and SD, will be computed for demographic characteristics, QoL, and parental outcomes (confidence, feeding, and teaching assessment using KPSC, NBRI score). Independent sample t-test, paired test, and repeated measure ANOVA will be used to compare within and between-subject effects. Multilevel logistic regression will also be performed for categorical outcomes to observe the effectiveness after controlling for the confounding factors. P≤0.05 will be considered significant.

In qualitative analysis, interviews will be transcribed verbatim and analyzed using software MAXQDA. A coding structure based on the interview guide will be iteratively developed to label specific interview content. The transcripts will be coded, and the two team members will discuss the results and resolve any disagreements in coding by consensus.

Translations, validation, translation, and adaptation of content

In the first round of validation, a group of stakeholders, including healthcare providers, teachers, ECD educators, parents, and grandparents, will be invited to assess the content of the intervention pack. All the relevant feedback will be incorporated into the final version of the curriculum. In the second round, two core groups from the field sites of Chitral and Afghanistan will be invited to explore the potential changes required in the curriculum. In the third round, the curriculum will be sent to the relevant healthcare facilities/community health workers for their rating and feedback. The curriculum was designed in English and will be translated into Urdu and Dari, for which a separate review by two local site co-investigators will be done to ensure its translation validation as per the context. Words that could not be part of literal translation will then be adapted to suit the cultural needs of both regions.

Delivery of the intervention

There will be a structured training whereby internationally trained master trainers in ECD and Keys to Infant Caregiving from Parent-Child Relationship Programs, at Barnard Center, University of Washington and internally certified on ECD PREP ObGyn Aga Khan University curriculum, will provide training to the field site investigators and coordinators, who will then cascade the training to the lady healthcare workers at each study setting. After receiving training, the trained healthcare providers will deliver the content in group-based teaching sessions. Each lady healthcare worker will be selected based on their prior experience in patient and family education by the healthcare facility lead and will undergo a four-day structured training program. Additionally, they will have monthly mentoring check-ins throughout the intervention and will be provided with constant support and random first-hand check-ins by the field coordinators, who will go and co-conduct and observe the session with them. The whole intervention will be closely supervised by the field co-investigators and the lead investigator. An assigned research specialist will conduct monthly meetings to provide the support needed at the field sites.

Ethical considerations

This proposal has received approval from the National Bioethics Commission (No.4-87/NBCR-986/23/323), Pakistan, the Aga Khan University ethical review committee (ERC # 2023-8343-24153), and the Institutional Review Board of Afghanistan (IRB Code No: A-8-24-445). Informed written consent will be sought from all the participants. They will be informed about all the modalities involved in the research project, their rights, and, in no form, will there be names or any identifiable information that will be sought or recorded. All forms will have a unique participant code. All the data will be kept confidential. The data collectors will collect the forms, seal the forms, and send them to the main institution. Additionally, the data will be kept in lock and key. Until the forms are sent here, all the data on the field sites will also remain in a locked drawer.

## Results

This study is currently in progress. Results will be disseminated between December 2025 and March 2026.

## Discussion

Responsive parenting is an art; parents need to be ready to welcome the new soul enter in the family with enough knowledge and skills; and a number of parents do not have enough information to handle the challenges related to breastfeeding and childcare and how to stimulate the newborn that optimize brain development and other lifelong trajectories of health and learning [27,28]. Parents living in rural settings and remote areas are more vulnerable due to the scarcity of scientific knowledge on responsive caregiving [28]. There is an urgent need to develop evidence-based ECD interventions that address the current-day challenges of parenting.

Moreover, primary healthcare centers in far-flung areas serve as a nucleus for multiple health interventions because of affordability and accessibility. Fostering children's physical, social, and emotional growth and developmental tracking is the primary objective of the primary health centres. These health facilities become a vital hub to deliver ECD parenting interventions to the mother who delivered at a health facility [29]. Evidence about the integration of ECD services in primary healthcare settings is effective and efficient [30]. However, the current ECD study will be unique in type and will be implemented through primary healthcare settings in Pakistan and Afghanistan. The study is implementation research in which the feasibility and adaptability of newborn parenting interventions in the primary healthcare settings will be explored. The primary healthcare centres are mostly run by LHVs and midwives. This research will allow building the capacity of the healthcare providers on the ECD newborn responsive caregiving package. Qualitative and quantitative arms of the study allow the researcher to explore the readiness of healthcare providers and the readiness of the system for ECD parenting intervention, opportunities and challenges faced by parents, and community stakeholders. This study will provide the evidence for ECD integration into primary healthcare settings in Pakistan and Afghanistan. This study will provide a culturally sensitive, inclusive framework of sustainability and scalability of newborn parenting packages in primary healthcare settings of the resource-poor communities.

The findings of this study will contribute to developing strategic guidelines appropriate to implement newborn ECD parenting interventions in primary health settings of resource-constrained areas, essential components of healthcare staff training, and determining the potential effectiveness and adaptability of ECD newborn parenting education programs in low- to middle-income countries such as Pakistan and Afghanistan. The study will also contribute to highlighting the potential enablers present in the healthcare system and policy documents that promote ECD service delivery and system barriers that hinder the smooth implementation of newborn ECD responsive parenting education programs to promote maternal and child health and development outcomes. Furthermore, the findings of this study will further contribute to building evidence-based ECD literature on newborn parenting intervention, hereby encouraging healthcare providers, policy makers, and public health implementers to design holistic ECD service packages. Additionally, the study's identification of implementation enablers and barriers will guide advancements in care providers and healthcare staff training on newborn responsive caregiving, the development of policies that support ECD newborn responsive parenting, and contribute to optimal maternal and child health outcomes. Furthermore, the study will add to growing literature evidence on newborn responsive caregiving and thereby encourage attempts to include ECD into maternity and child health services.

Results from the ECD Parenting Education study are due by the end of 2025; the findings of the study will be disseminated through dissemination seminars at the field site and advocacy conferences, panel discussions on national and international forums, and publication in peer-reviewed journals.

Plausible limitations and risk mitigation

There are possible limitations of this study. A convenience sampling technique will be used, which might lead to low generalizability and underrepresentation of the targeted population. The reason for using this sampling technique because it depends on the volume of deliveries in healthcare facilities in both regions. This seems to be best suited within the context of adaptation and exploring the implementation impact. However, a randomized controlled trial is recommended in the future to see the results of the intervention on a large scale. Challenges we might face due to having two different geographical settings. One geographical setting has a post-crisis conflict recovery and changes of regime in the region. In the second setting, where climate crises are happening, that might limit to achievement of the target sample size.

## Conclusions

This protocol aims to conduct a before-and after (quasi-experimental design for this arm of the study) and implement a newborn parenting education program in primary and secondary healthcare settings in low- and middle-income countries such as Pakistan and Afghanistan, where early childhood development and responsive parenting education in healthcare settings are least prioritized. The findings of this study, through its data, will inform policy makers and other key stakeholders in understanding the significance and nuances of integrating the early childhood responsive parenting education program in the existing maternal and child health system, particularly in primary and secondary healthcare settings.

## Appendices

Study Intervention Content Links

Early Childhood Development Newborn Responsive Caregiving: https://akflearninghub.org/document/early-childhood-development-newborn-responsive-caregiving/

AKU Website: https://www.aku.edu/mcpk/obs-gyn/Pages/ecd-prep.aspx
